# Alleviation of Oxidative Damage Induced by CaCl_2_ Priming Is Related to Osmotic and Ion Stress Reduction Rather Than Enhanced Antioxidant Capacity During Germination Under Salt Stress in Sorghum

**DOI:** 10.3389/fpls.2022.881039

**Published:** 2022-04-29

**Authors:** Xiaofei Chen, Ruidong Zhang, Bang Li, Tong Cui, Chang Liu, Chunjuan Liu, Bingru Chen, Yufei Zhou

**Affiliations:** ^1^College of Agronomy, Shenyang Agricultural University, Shenyang, China; ^2^Crop Research Institute, Anhui Academy of Agricultural Sciences, Hefei, China; ^3^Institute of Economic Crop, Shanxi Academy of Agricultural Sciences, Fenyang, China; ^4^Institute of Crop Germplasm Resources, Jilin Academy of Agricultural Sciences, Changchun, China

**Keywords:** sorghum germination, seed priming, saline stress, osmotic adjustment, ion transport, calcium signaling

## Abstract

Seed germination is the sensitive period to salt stress. Calcium chloride (CaCl_2_) has been proved as an effective priming agent which can promote the sorghum germination under salt stress. However, there are few reports on CaCl_2_ priming to improve the salt tolerance during seed germination. The present study investigated the effects of CaCl_2_ priming on sorghum germination, antioxidant metabolism, osmotic regulation and ion balance under salt stress (150 mM NaCl). The results revealed that the salt stress inhibited the elongation of mesocotyl and root and reduced the germination rate of sorghum. While CaCl_2_ priming significantly promoted the elongation of mesocotyl and root, and increased the germination rate of sorghum under salt stress. CaCl_2_ priming notably increased the content of osmotic substances in mesocotyl and root of sorghum under salt stress, and increased the relative water content in these tissues. CaCl_2_ priming decreased Na^+^ content and increased K^+^, Ca^2+^ contents and the K^+^/ Na^+^ in mesocotyl and root, such effects might be induced by up-regulating the expression of *NHX2*, *NHX4*, *SOS1*, *AKT1*, *AKT2*, *HKT1*, *HAK1*, and *KUP*. CaCl_2_ priming reduced the antioxidant enzymes activities and related gene expression compared with untreated sorghum seeds under salt stress. In short, CaCl_2_ priming improved sorghum germination by enhancing osmotic regulation and ion balance instead of antioxidant enzyme activity. However, the molecular mechanisms of Ca^2+^ signaling induced by CaCl_2_ priming in association with the enhanced germination in primed sorghum seeds under salt stress need to be addressed in future studies.

## Introduction

Worldwide, land salinization is a serious environmental problem. More than 6% of world land area is saline and 20% of irrigated lands are affected by salinity (Song and Wang, 2014; Yuan et al., 2016). sorghum is a highly resistant crop and has received extensive attention due to its huge production potential that can meet the challenges of global food security (Hao et al., 2021). However, sorghum is very sensitive to salt during the germination stage. Salt stress will affect emergence and establishment of early seedlings, and reduce the final yield (Zhu et al., 2018). Seed germination is critical to successful crop production (Hajihashemi et al., 2020). There are several reports about salt stress delaying and inhibiting seed germination and plant growth (Ning et al., 2019; Sheteiwy et al., 2020). Therefore, improving seed germination under salt stress is of great significance for improving the emergence and production of sorghum in saline soil.

Seed priming is a convenient, inexpensive, and low-risk means that can effectively improve the emergence of crops in saline soil. It is also the most commonly used technical intervention for farmers to improve seed germination (Hussain et al., 2018). Seed priming refers to initially exposing the seed to a promoter that makes the seed more resistant to future stress (Tanou et al., 2012). Partial hydration of seeds in a specific environment occurs during priming (Giri and Schillinger, 2003). Priming activates positive metabolic processes for seeds to resist different abiotic stresses (Jisha et al., 2013). Previous studies revealed that seed priming can significantly alleviate the inhibition of salt stress on crop seed germination (Shah et al., 2020; Chen et al., 2021).

Environmental changes lead to frequent occurrences of abiotic stress and severe damage to plant health (Abdoulaye et al., 2019). Changes of substances metabolism and gene expression occur in plant tissue and cell under abiotic stress especially salt stress (Ibrahim, 2016; He et al., 2017; Gao et al., 2020). The effects of salt stress on crops involve primary stress and secondary stress. The former includes osmotic stress and ion stress, and the latter includes the production of reactive oxygen species (ROS) (Wani et al., 2018; Yang and Guo, 2018). Under salt stress, high concentrations of salt ions reduce the free energy of water and the resulting decline in the water potential of the surrounding environment leads to osmotic stress in plant growth. Therefore, plants experience difficulty in absorbing water, leading to plant water deficit. The osmotic regulators, such as proline, amino acids, soluble sugars, and soluble proteins, plays a vital role during seed germination under salt stress, which can reduce cell osmotic potential and promote cell water absorption and seed germination (Ibrahim, 2016). Various studies suggest that seed priming can enhance the ability of osmotic adjustment in plants under salt stress (Tabassum et al., 2017; Bajwa et al., 2018; Rehman et al., 2021).

High concentration of sodium ions is the main cause of salt induced ion stress (Ismail et al., 2014). Seed germination is inhibited by salt due to the toxic effects of sodium ions on embryo activity, including structural damage to macromolecular substances and the cell membrane (Daszkowska-Golec, 2011). Salt is absorbed by roots and transported to all parts of the plant. The accumulation of sodium ions in cytoplasm will affect the absorption of other ions, such as potassium ions, which in turn affects the normal physiological metabolism of plants. For instance, potassium ions are crucial for the catalytic activity of many enzymes (Fu and Luan, 1998; Lazof and Bernstein, 1998; Munns and Tester, 2008). Calcium is an important component of cell structure and involved in cell elongation and cell division processes. It plays a significant role in regulating nutrient absorption across cell membranes and improving water absorption (Summart et al., 2010). In addition, Ca^2+^ can alleviate the negative effects of Na^+^ on plant growth (Gobinathan et al., 2009). Therefore, maintaining the ion homeostasis in plant cells under salt stress is essential to improve salt tolerance. The role of seed priming in maintaining ion homeostasis under salt stress has been reported in many literatures (Bajwa et al., 2018; Mahboob et al., 2019; Wang et al., 2019). However, there are fewer reports on the homeostasis of calcium and sodium ions than those on the homeostasis of potassium and sodium ions. Secondary stress induced by salt, such as excessive accumulation of ROS, can cause serious damage to cell structure and macromolecular substances (Genisel et al., 2015; Yang and Guo, 2018). An evolved complex antioxidant system in plants can remove excess ROS and reduce cellular oxidative damage. The ROS scavenging system includes non-enzymatic antioxidants, such as proline, and antioxidant enzymes, including the superoxide dismutase (SOD), peroxidase (POD), catalase (CAT), and ascorbate peroxidase (APX) (El-Beltagi et al., 2020). Antioxidant substances activity and synthesis are affected by abiotic stress (Sheteiwy et al., 2021; Yang et al., 2021). Although most studies have indicated that seed priming alleviates salt stress damage through enhanced antioxidant activity (Jiang et al., 2020; Soliman et al., 2020), a few studies have shown that seed priming did not enhance antioxidant enzyme activities. For example, Genisel et al. (2015) reported that cysteine priming reduced the production of ROS under salt stress and reduced oxidative damage, but the activity of antioxidant enzymes was significantly decreased. It was inferred that the reduction of cysteine alone led to a decrease in the demand for the antioxidant system. Therefore, further studies are needed to determine whether seed priming enhances the comprehensive resistance mechanism induced by salt stress, since the secondary stress is fundamentally caused by the primary stress.

Calcium chloride has been proved to be the most effective priming agent to promote sorghum germination under salt stress (Chen et al., 2021), but the physiological effects of calcium chloride are not well understood. The purpose of this study was to investigate (1) the effect of calcium chloride on osmotic adjustment, ion homeostasis. and antioxidant metabolism during sorghum germination under salt stress; and (2) the metabolic pathway(s) through which calcium chloride plays a key role in alleviating the inhibition of salt stress on sorghum germination.

## Materials and Methods

### Seed Material and Experimental Design

Sorghum (*Sorghum bicolor* (L.) Moench) seeds of Liaoza15 used in this study were provided by the Liaoning Academy of Agricultural Sciences. Uniform seeds were disinfected with 5% NaClO solution for 5 min, then rinsed with distilled water three to five times, and wiped the surface water. Seeds were primed with distilled water or CaCl_2_ solution for 12 h at 25°C in the dark, and the ratio of seed weight to solution volume was 1:5 (w/v). Then, the seeds were dried to the original moisture content at room temperature (25°C) after wiping the surface moisture. Preliminary test results showed that CaCl_2_ solution with a concentration of 6 g.L^–1^ had the best priming effect. The no primed seeds were, respectively, cultured under distilled water (NPN) or 150 mM NaCl (NPS). The seeds subjected to hydro-priming (HPS) and CaCl_2_ priming (CaPS) were both cultured under NaCl stress. The seeds were incubated in a Petri dish covered with double-layer filter paper, and 10 mL of distilled water or NaCl solution was added to each Petri dish. The Petri dish was placed in a completely dark climate-controlled incubator at a temperature of 25 ± 1°C and 70% humidity. Each treatment was repeated three times, and each replicate contained 50 seeds. The number of germinated seeds were counted on the 7th day and the germination rate was calculated. The germination was determined to have occurred once the radicle length was equal to the seed length and the bud was half the seed length. The mesocotyl length, root length, fresh weight, and dry weight were measured. The mesocotyl and root tissues were quickly placed in liquid nitrogen and stored at −80°C for the determination of physiological parameters.

### Germination Rate and Growth Parameters

On the 7th day after germination, 10 plants were randomly selected from each replicate to determine the mesocotyl length, root length, fresh weight, and dry weight. The calculation formula for germination rate is as follows:


Germinationrate(%)=Total⁢number⁢of⁢germinated⁢seeds⁢on⁢day⁢ 7Total⁢number⁢of⁢seeds×100%

### Moisture Status and Osmotic Adjustment Substances

Fresh mesocotyl and root tissues were weighed (FM), quickly immersed in distilled water for 24 h, the surface was wiped, the swollen mass (TM) weighed, and finally dried in an oven at 80°C to constant weight (DM). The formula for determining relative water content (RWC) is as follows (Hayat et al., 2007):


$$\text{RWC}\left(\%\right)=\frac{\mathrm{FM}-\mathrm{DM}}{\mathrm{TM}-\mathrm{DM}}\times 100\%$$

Proline content was determined using ninhydrin method (Bates et al., 1973). Soluble protein content was determined using coomassie brilliant blue (CCB) based on the method of Guzel and Terzi (2013). Soluble sugar content determination was conducted according to the method of Quan et al. (2004). Free amino acid content was measured according to the method described by Zhang et al. (2015).

### Determination of Na^+^, K^+^, Ca^2+^ Content

Concentrated sulfuric acid of 10 mL was added to 0.1 g of the dried sample and digested in a digestion furnace at 320°C for 30 min. During digestion, 30% hydrogen peroxide solution was added twice until the digestion solution was pellucid. The digestion solution was made up to 100 mL with distilled water and the flame photometer (AP1200, AOPU analytical instrument, Shanghai, China) was used for ion content determination. The ion content was calculated using the standard curves of Na^+^, K^+^, and Ca^2+^ (Shi et al., 2020).

### Reactive Oxygen Species and Membrane Damage

The determination of hydrogen peroxide content was carried out based on the method of Velikova et al. (2000). Preparation of diaminobenzidine (DAB) staining solution: distilled water was added to an appropriate amount of DAB to make the concentration 1 mg.mL^–1^. Adjusting pH to 3.8 with concentrated hydrochloric acid dropwise to dissolve DAB, then the pH was adjusted to 5.8 with sodium hydroxide before use. Mesocotyl and root were incubated in DAB solution in the dark at 25°C for more than 8 h.

The determination of O^2–^ was determined by referring to Tian et al. (2003). Preparation of nitrotetrazolium blue chloride (NBT) staining solution: an appropriate amount of NBT was dissolved in 10 mM potassium phosphate buffer (pH 7.6) to 0.5 mg.mL^–1^ and reserved at 4°C in the dark. The plant material was placed in the NBT solution for 3 h in the dark at room temperature. The electrolyte leakage rate was conducted according to Hniličková et al. (2019). The electrolyte leakage (EL) is determined according to the following formula:


$$\mathrm{EL}\left(\%\right)=\frac{\mathrm{EC1}}{\mathrm{EC2}}\times 100$$

Where the EC1 refers to the initial conductivity and EC2 represents the final conductivity.

Malondialdehyde (MDA) content determination is based on the method of Zhang et al. (2007).

### Antioxidant Enzyme Activity and Electrophoresis

The extraction of antioxidant enzymes was conducted using the method of Gogorcena et al. (1995). This antioxidant extract can be used to determine its activity. The SOD activity was determined using the method of Beyer and Fridovich (1987). The POD activity was measured according to Fielding and Hall (1978). The determination of CAT activity refers to the method of Aebi (1984). The determination of APX activity refers to the method of Amako et al. (1994).

Antioxidant enzyme isoenzymes were separated on a 1 mm thick polyacrylamide gel (3% concentrated gel, 7% separating gel). Detailed electrophoresis methods refer to the description of Jin et al. (2009). The SOD isoenzyme was stained using the o-dianisidine staining method. The staining solution comprised 2 mM o-dianisidine, 0.1 mM riboflavin, 20% ethanol, and 10 mM pH 7.2 phosphate buffer, and the gel was immersed in the staining solution for 30 min, taken out, and rinsed with distilled water. The gel was then immersed in phosphate buffer solution and exposed to a fluorescent lamp for 30 min, and pictures were taken after the bands became clear. Benzidine staining method was adopted for POD isoenzyme staining. The gel was stained with 0.1% benzidine in acetate buffer (100 mM, pH 4.5) containing 2 mM H_2_O_2_, and then immersed in 7% (v/v) acetic acid to terminate the reaction. Starch method was adopted for CAT isoenzyme staining; 1% soluble starch was boiled until colorless and transparent. The gel was soaked in starch solution for 1 h and 40 min. Then the starch solution was discarded, 0.5% H_2_O_2_ was added to the gel for 1 min, and rinsed with distilled water. Finally, 0.5% potassium iodide solution was added. The background turned blue at 25°C and the areas with enzyme activity were colorless and transparent. APX staining was performed as follows: the gel was soaked in 50 mM PBS (pH 7.0) containing 2 mM ascorbic acid for 30 min, incubated for 20 min with 50 mM PBS containing 4 mM ascorbic acid and 2 mM H_2_O_2_, and then the gel was washed with 100 mM PBS (pH 7.5). The staining solution contained 28 mM TEMED and 2.45 mM NBT PBS (pH 7.8). The gel in staining solution was shaken gently and rinsed with distilled water. The color development was observed and photographs were taken.

### RNA Extraction

Fresh samples were thoroughly ground with TRNzol Universal reagent in liquid nitrogen. The grinding process was carried out rapidly, preferably not more than 1 min, using 0.1 g of sample used 1 mL TRNzol Universal reagent. The homogenized sample was stored at 25°C for 10 min to make the nucleic acid-protein complex separated. The sample was centrifuged at 4°C and 12,000 rpm (approximately 13,400 × *g*) for 10 min. Chloroform (0.2 mL) was added to each 1 mL of TRNzol Universal reagent, and was vigorously shaken for 15 s, and stored at 25°C for 3 min. Then the sample was centrifuged at 12,000 rpm (∼13,400 × *g*) at 4°C for 15 min and separated into three layers: pink organic phase, middle layer, and upper colorless water phase. The water phase (about 500 μL) containing RNA was poured into a new centrifuge tube and an equal volume of isopropanol was added. After mixing, it was allowed to stand at 25°C for 10 min, then centrifuged at 4°C, 12,000 rpm (∼13,400 × *g*) for 10 min, and the supernatant was discarded. The RNA precipitate is usually invisible before centrifugation. The RNA forms a gel-like precipitate on the side and bottom of the tube after centrifugation. Then, 1 mL of 75% ethanol was used to wash the precipitate. For every 1 mL TRNzol Universal reagent, at least 1 mL of 75% ethanol should be used to disperse the precipitate, then centrifuged at 4°C and 10,000 rpm (∼9,391 × *g*) for 5 min. We poured out the liquid, but were careful not to pour out the precipitate. The remaining liquid was centrifuged briefly and was carefully aspirated without disturbing the precipitate. The sample was then dried at 25°C for 2–3 min (we were careful to avoid over-drying as it is difficult to dissolve the RNA after complete drying), according to the needs of the experiment, 30–100 μL RNase-free ddH_2_O was added to the sample and mixed by repeated pipetting to fully dissolved the RNA. The gene ID and primer information used for qRT-PCR are shown in Table 1.

**TABLE 1:** Primer used for qRT-PCR analysis.

Gene name/Gene ID	Forward primer	Reverse primer
*SOD2*/110432872	GACAGCCAGATCCCTCTCAC	ATGATACCACACGCAACACG
*SOD4A*/110431820	TCAGTGTTACCGACAGCCAG	TGATCCCACAAGCAACACGG
*POD1*/8086300	GTGGAAGGAGAAATTCGCGG	GTAGCCCTCTTCATCGACGG
*POD2*/8055467	GCGCATCTTCTTCCACGACT	TGTCTCGGATGCTCTCGATG
*CAT1*/8069231	TGCTCAGTTCGACAGGGAAC	CTTCCACGCTCATGCACAAC
*CAT2*/110430724	ATCCAGACCATGGACCCTGA	CTCGTTCTCGGCGAAGAAGT
*CAT3*/8068221	GATCGGCAGGAGAGGTTTGT	GCTTCATGTTGAGCCGTGTG
*NHX2*/8074408	ATGTCGGGGATTTTCTCGCT	GACGTGGCATCGTTCACAAC
*NHX4*/8078249	AGTTTCGGCAGGCTGCTATT	AGCCTTGGTGTCTCGTCTTG
*AKT1*/8058236	TGAACCATGAAGACGCCGAA	AAGGCCTTTCGAGAGCAGAC
*AKT2*/8058492	AAGCCATCGTCGGTGAGAAA	ATTGATGCCAGCCTCCTCAG
*SOS1*/Sb08g023290	TGAAGACCGACAACCTG	GCTTCCACCTGATACCT
*HKT1*/Sbrt06g	ATCGCCATCTGCATCACC	GCCTCCCAAAGAACATCACA
*HAK1*/8072046	TCAGAACGACCACCAGCATC	GCCGAACACCACGTAGAAGA
*KUP*/8059603	GAGGACGCAACGGTATCCAA	ACAGAGATTGCTGGCGTCAA

### Statistical Analysis

One-way analysis of variance (ANOVA) was carried out using SPSS 18.0 software (SPSS Inc., Chicago, IL, United States). Duncan’s multiple range test was used to assess significant differences (*p* < 0.05) among treatments using SPSS 18.0. Graph Pad Prism 8 (Graph Pad Software Inc., San Diego, CA, United States) is used for graphing, and the data in graphs are all expressed as mean ± standard deviation.

## Rusults

### Germination Parameters

**FIGURE 1:**
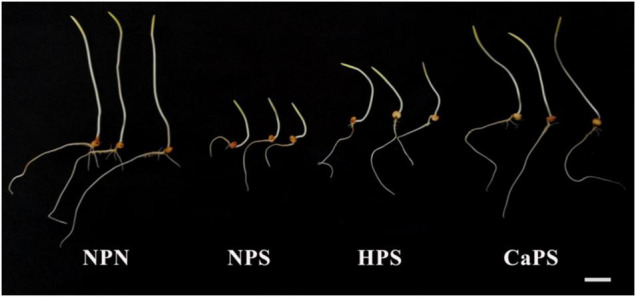
Effect of CaCl_2_ priming on sorghum germination phenotype under salt stress.

The germination phenotypes of different treatments are shown in Figure 1. Salt stress inhibited the seed germination, which manifested as reduction in shoot and root length. Hydro-priming and CaCl_2_ priming promoted the germination of sorghum seeds under salt stress. The shoot and root length were significantly increased and the effect of CaCl_2_ priming was significantly better than that of hydro-priming.

Salt stress significantly reduced the germination rate, mesocotyl length, mesocotyl fresh weight, mesocotyl dry weight, root length, root fresh weight, and root dry weight by 21.53, 67.07, 66.49, 46.25, 74.38, 74.14, and 35.40%, respectively (Table 2). There was no significant difference between HPS treatment and NPS treatment in terms of germination rate and root dry weight. Mesocotyl length, mesocotyl fresh weight, mesocotyl dry weight, root length, and root fresh weight in HPS were significantly increased by 96.35, 95.26, 34.56, 164.94, and 154.97%, respectively, compared with NPS treatment. The germination rate, mesocotyl length, mesocotyl fresh weight, mesocotyl dry weight, root length, root fresh weight, and root dry weight of CaPS treatment were significantly increased by 27.45, 169.79, 206.05, 82.35, 219.48, 258.94, and 16.44%, respectively, compared with NPS treatment. It is observed that CaCl_2_ priming could effectively improve seed germination under salt stress.

**TABLE 2:** Effect of CaCl_2_ priming on seed germination traits in sorghum under salt stress.

Treatment	GR(%)	ML(cm)	MFW(mg)	MDW(mg)	RL(cm)	RFW(mg)	RDW(mg)
NPN	86.67 ± 8.33a	11.66 ± 0.51a	81.82 ± 9.90a	6.33 ± 0.82a	12.02 ± 0.97a	35.04 ± 5.38a	2.83 ± 0.15a
NPS	68.00 ± 10.58b	3.84 ± 0.40d	27.42 ± 4.81c	3.40 ± 0.37c	3.08 ± 0.67d	9.06 ± 1.24c	1.83 ± 0.15c
HPS	80.00 ± 4.00ab	7.54 ± 0.59c	53.54 ± 8.40b	4.58 ± 0.29b	8.16 ± 0.92c	23.10 ± 3.82b	1.80 ± 0.14c
CaPS	86.67 ± 2.31a	10.36 ± 0.57b	83.92 ± 6.79a	6.20 ± 0.61a	9.84 ± 0.49b	32.52 ± 5.27a	2.13 ± 0.13b

*GR, germination rate; ML, mesocotyl length; MFW, mesocotyl fresh weight; MDW, mesocotyl dry weight; RL, root length; RFW, root fresh weight; RDW, root dry weight. Different lowercase letters within one column indicate significant difference (p < 0.05, Duncan’s multiple range test).*

### Relative Water Content

**FIGURE 2:**
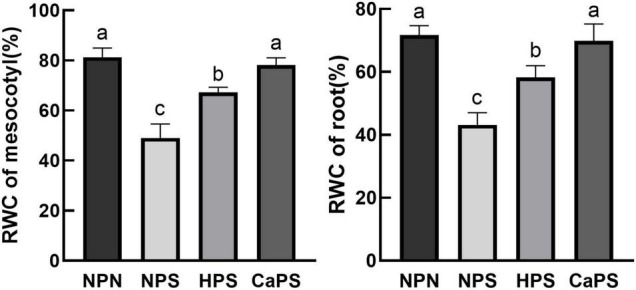
Effect of CaCl_2_ priming on relative water content of sorghum tissue under salt stress. RWC, relative water content.

Under salt stress, the relative water content of mesocotyl and root was significantly reduced by 39.70 and 39.76%, respectively. Compared with NPS treatment, the relative water content of mesocotyl and root was significantly increased by 37.32 and 35.00% in HPS treatment. Compared with NPS treatment, the relative water content of mesocotyl and root in CaPS treatment were significantly increased by 59.31 and 61.81%, respectively (Figure 2). These results indicated that CaCl_2_ priming significantly improved water status under salt stress.

### Accumulation of Osmotic Adjustment Substance

**FIGURE 3:**
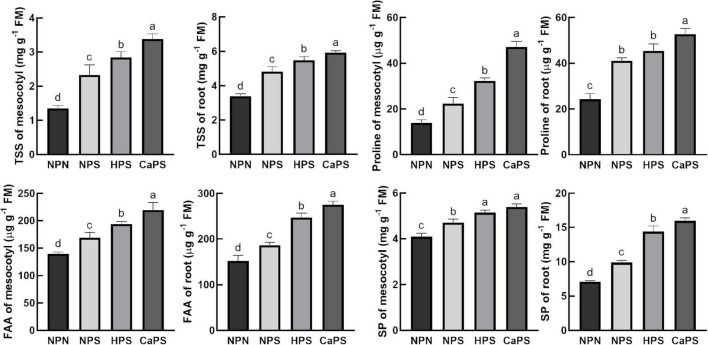
Effect of CaCl_2_ priming on osmotic adjustment substances in sorghum tissue under salt stress. TSS, total soluble sugar; FAA, free amino acid; SP, soluble protein.

Under salt stress, the content of soluble sugar, proline, free amino acid, and soluble protein in mesocotyl were significantly increased by 72.21, 60.50, 21.13, and 15.05%, respectively (Figure 3). The content of soluble sugar, proline, free amino acid, and soluble protein in root, respectively, increased 42.26, 68.79, 22.53, and 39.30%. Compared with NPS treatment, the soluble sugar, proline, free amino acid, and soluble protein content in mesocotyl were significantly increased by 22.21, 44.31, 14.64, and 9.25% in the HPS treatment, while they significantly increased in the root by 13.57, 10.71, 32.60, and 45.56%, respectively. Compared with NPS treatment, the content of soluble sugar, proline, free amino acid, and soluble protein increased significantly in CaPS treatment by 45.43, 110.69, 30.04, and 14.52%, and increased by 23.04, 28.61, 47.99, and 61.47% in root, respectively. Changes in these osmotic adjustment substances illustrated that CaCl_2_ priming induced improvement of osmotic adjustment ability.

### Na^+^, K^+^, and Ca^2+^ Content

Under salt stress, Na^+^ content in mesocotyls and roots were significantly increased by 53.15 and 125.53%, respectively, while K^+^ content was significantly increased by 23.86 and 80.46%, respectively. Ca^2+^ content had no significant change under salt stress. K^+^/Na^+^ in mesocotyl and root were significantly reduced by 17.78 and 18.75%, while Ca^2+^/Na^+^ were significantly reduced by 42.99 and 58.14%, respectively. Compared with NPS, the Na^+^ content of mesocotyl and roots in HPS treatment was significantly reduced by 18.32 and 26.92%, respectively, and there was no significant difference in K^+^ content. Ca^2+^ content was significantly increased by 49.65 and 29.53%, and K^+^/Na^+^ was significantly increased by 34.46 and 45.56%, respectively. Ca^2+^/Na^+^ was increased significantly by 84.79 and 77.74%, respectively. Compared with NPS treatment, the Na^+^ content of mesocotyl and root was significantly reduced by 32.34 and 28.15% in CaPS treatment, respectively. K^+^ content was significantly increased by 34.54 and 37.81%, Ca^2+^ content was significantly increased by 170.04 and 113.50%, and K^+^/Na^+^ was significantly increased by 98.21 and 90.96%, Ca^2+^/Na^+^ was increased significantly by 302.27 and 197.74%, respectively (Table 3). These results showed that CaCl_2_ priming significantly improved ion balance under salt stress.

**TABLE 3:** Effect of CaCl_2_ priming on ion content in sorghum tissue under salt stress.

Tissue	Treatment	Na^+^ (mg g^–1^ DM)	K^+^ (mg g^–1^ DM)	Ca^2+^ (mg g^–1^ DM)	K^+^/Na^+^	Ca^2+^/Na^+^
Mesocotyl	NPN	11.50 ± 0.28c	5.23 ± 0.42c	0.62 ± 0.03c	0.45 ± 0.04b	0.05 ± 0.002b
	NPS	17.61 ± 1.08a	6.47 ± 0.41b	0.55 ± 0.11c	0.37 ± 0.04c	0.03 ± 0.005c
	HPS	14.39 ± 1.12b	7.10 ± 0.87b	0.82 ± 0.07b	0.50 ± 0.08b	0.06 ± 0.006b
	CaPS	11.92 ± 0.44c	8.71 ± 0.35a	1.48 ± 0.09a	0.73 ± 0.04a	0.12 ± 0.011a
Root	NPN	21.56 ± 0.13c	3.51 ± 0.37c	1.37 ± 0.20bc	0.16 ± 0.02b	0.06 ± 0.010b
	NPS	48.63 ± 2.28a	6.34 ± 0.39b	1.29 ± 0.11c	0.13 ± 0.01c	0.03 ± 0.001d
	HPS	35.58 ± 1.98b	6.73 ± 0.70b	1.68 ± 0.23b	0.19 ± 0.03b	0.05 ± 0.006c
	CaPS	34.94 ± 0.62b	8.73 ± 0.97a	2.76 ± 0.12a	0.25 ± 0.01a	0.08 ± 0.002a

*Different lowercase letters within one column indicate significant difference (p < 0.05, Duncan’s multiple range test).*

### Accumulation of Reactive Oxygen

**FIGURE 4:**
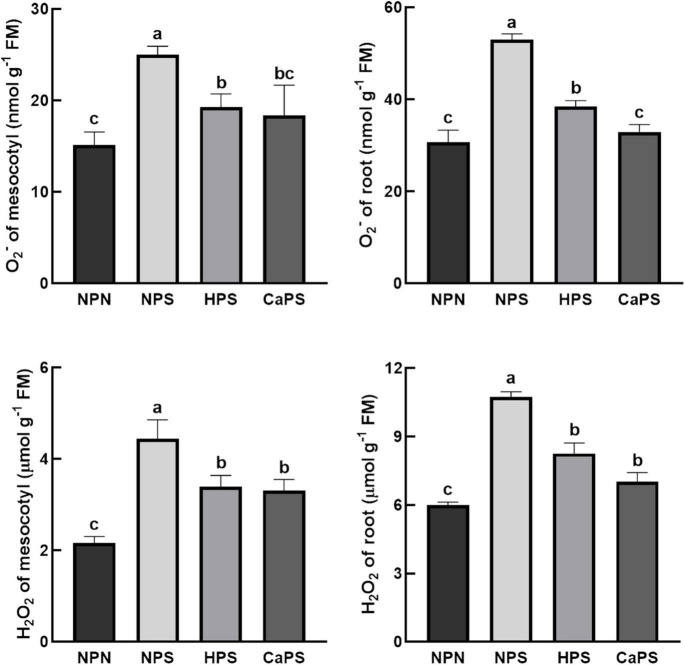
Effect of CaCl_2_ priming on reactive oxygen in sorghum tissue under salt stress.

**FIGURE 5:**
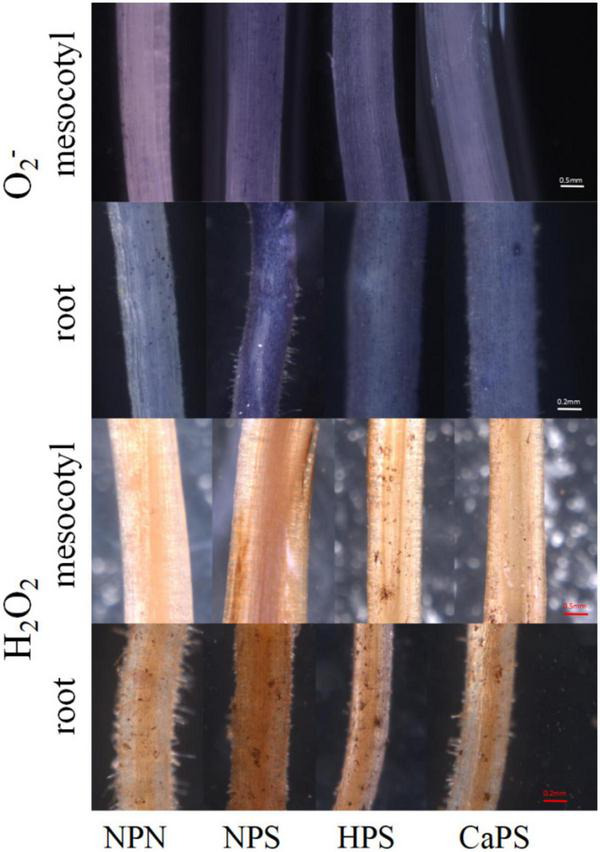
Staining of ROS in sorghum mesocotyl and root.

Salt stress notably increased the content of O^2–^ and H_2_O_2_ in mesocotyl and root, which was increased by 65.15 and 105.40% in mesocotyl, and 72.69 and 79.11% in root, respectively (Figure 4). Compared with NPS, both hydro-priming and CaCl_2_ priming treatments significantly reduced the level of reactive oxygen species. The content of O^2–^ and H_2_O_2_ in the mesocotyl were reduced by 22.86 and 23.61% in HPS treatment, and by 27.48 and 23.09% in root, respectively. The content of O^2–^ and H_2_O_2_ in mesocotyl were reduced by 26.47 and 25.56%, respectively, in CaPS treatment, and by 37.89 and 34.52% in root, respectively. The results of reactive oxygen staining were also basically consistent with the quantitative results (Figure 5). It can be seen from these results that the ROS level was significantly reduced by CaCl_2_ priming.

### Membrane Damage

**FIGURE 6:**
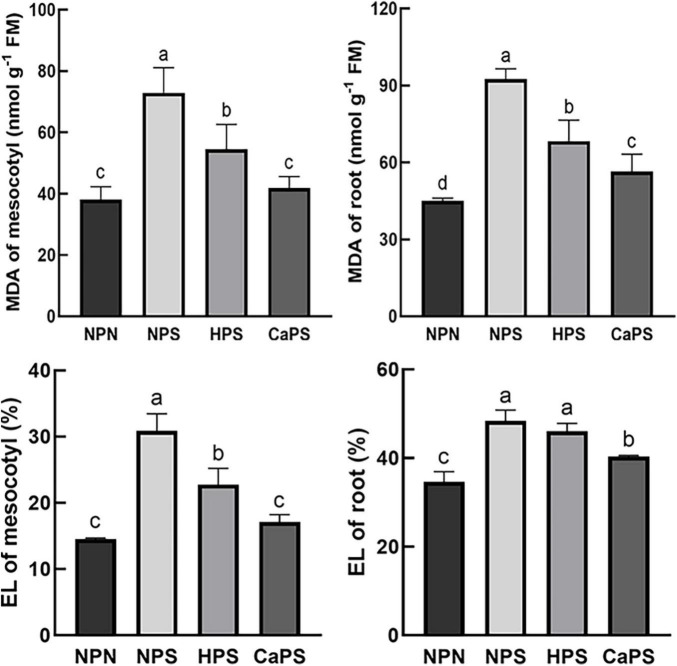
Effect of CaCl_2_ priming on MDA content and electrolyte leakage in sorghum tissue under salt stress. MDA, malondialdehyde; EL, electrolyte leakage.

Salt stress significantly increased MDA content and electrolyte leakage in the mesocotyl and root tissues (Figure 6). The MDA content increased significantly by 91.24 and 105.27%, and the electrolyte leakage increased by 112.83 and 39.85%, respectively. Compared with NPS, the MDA content of mesocotyl and root was significantly reduced by 25.26 and 26.20% in HPS treatment, while the electrolyte leakage of the mesocotyl was significantly reduced by 26.31% in HPS treatment. The MDA content of mesocotyl and root was significantly reduced by 42.67 and 39.05% in CaPS treatment, while electrolyte leakage was significantly reduced by 44.52 and 16.58% in CaPS treatment, respectively, indicating that CaCl_2_ priming could effectively protect plasma membranes under salt stress.

### Antioxidant Enzyme System

**FIGURE 7:**
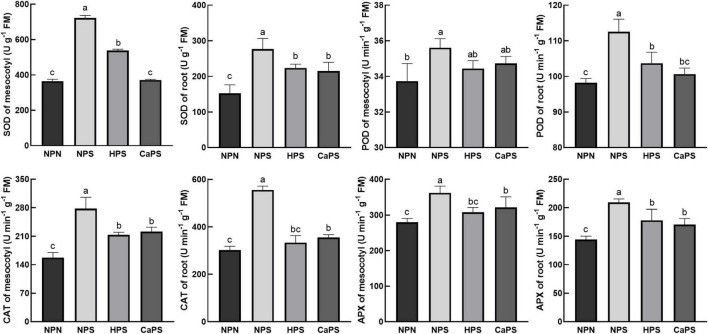
Effect of CaCl_2_ priming on antioxidant enzyme activities in sorghum tissue under salt stress.

**FIGURE 8:**
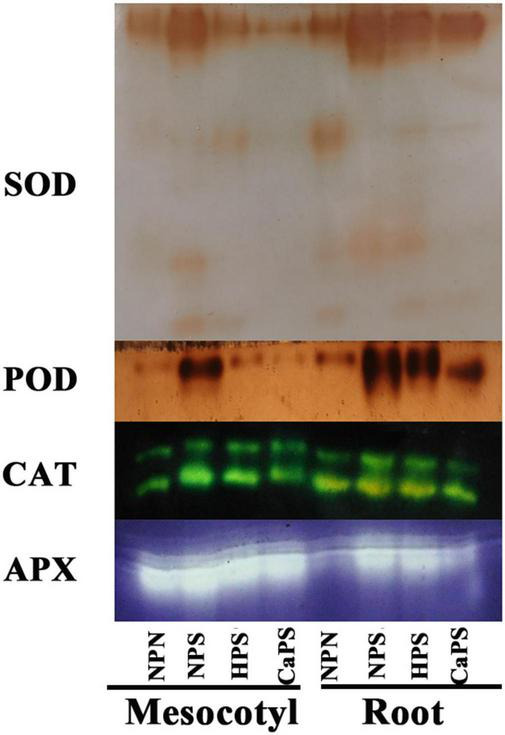
Staining of antioxidant enzymes in mesocotyl and root.

Salt stress significantly increased the activities of antioxidant enzymes in sorghum tissues (Figure 7). The activities of SOD, POD, CAT, and APX in mesocotyl were significantly increased by 97.84, 5.57, 76.10, and 29.40%, and increased by 81.56, 14.59, 83.37, and 44.86% in root. Compared with NPS treatment, the activities of SOD, CAT, and APX in mesocotyl were significantly reduced by 25.51, 23.12, and 14.97% in HPS treatment, and the activities of SOD, POD, CAT, and APX in roots were significantly decreased by 19.26, 7.85, 39.82, and 14.78%, respectively. Compared with NPS treatment, the activities of SOD, CAT, and APX in mesocotyl were significantly reduced by 48.59, 20.27, and 11.26% in the CaPS treatment, and the activities of SOD, POD, CAT, and APX in the roots were significantly decreased by 22.41, 10.56, 35.99, and 18.42%, respectively. The results of antioxidant enzyme staining are consistent with the quantitative results (Figure 8). These results suggested that seed priming did not further enhance antioxidant enzyme activity under salt stress.

### Gene Expression Level

**FIGURE 9:**
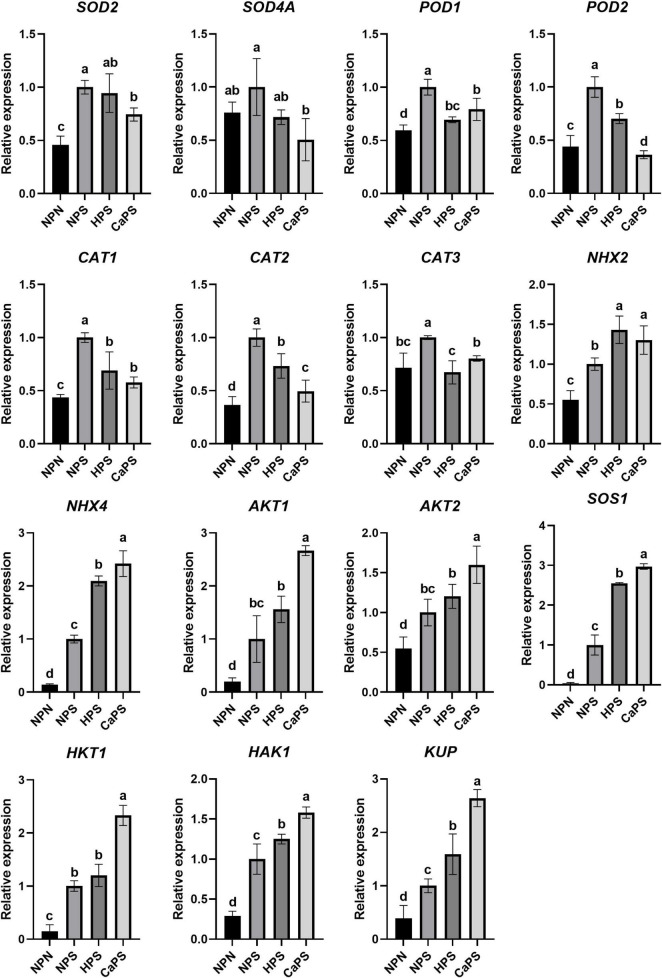
Effects of CaCl_2_ priming on the expression of antioxidant enzymes and ion transport genes in sorghum under salt stress.

Under salt stress, the expression of superoxide dismutase genes *SOD2*, peroxidase genes *POD1*, *POD2*, catalase genes *CAT1*, *CAT2*, and *CAT3* were significantly up-regulated, which were up-regulated by 118.47, 68.07, 126.45, 129.79, 172.19, and 39.40%, respectively. CaCl_2_ priming treatment reduced the expression of these genes (Figure 9). Compared with NPS, the expression levels were reduced by 25.74, 20.86, 63.53, 42.24, 50.38, and 19.66%, respectively. The expression level of *SOD4A* was significantly reduced by 49.45%. These results suggested that changes in antioxidant enzyme activities were correlated with gene expression levels. Under salt stress, the expressions level of Na^+^/H^+^ antiporter genes, *NHX2*, *NHX4*, *SOS1*, and K^+^ transporter genes, *AKT1*, *AKT2*, *HKT1*, *HAK1*, and *KUP* were up-regulated by 81.78, 624.86, 2134.87, 394.22, 86.41, 584.78, 242.53, and 158.84%, respectively. Compared with NPS, CaCl_2_ priming treatment further increased the expression of these genes, which were increased by 30.23, 142.05, 163.52, 166.51, 29.95, 133.36, 58.23, and 163.52%, respectively, indicating that CaCl_2_ priming enhanced the genes expression of ion transporter to resist ion stress.

## Discussion

Physiological and molecular changes of plant tissues and cells under abiotic stress affect plant growth and development (He et al., 2017; Gao et al., 2020; Basit et al., 2022). Salt stress negatively affects many growth processes of plants, such as seed germination rate, germination induction, and seedling establishment (Ning et al., 2019; Sheteiwy et al., 2020). Previous studies have shown that calcium plays a key role in plant response to abiotic stress (Niu et al., 2017), and in plant growth and development, including seed germination (Kong et al., 2015). This study found that salt stress significantly reduced the germination rate and growth. Hydro-priming and CaCl_2_ priming promoted germination under salt stress to varying degrees, and the effect of CaCl_2_ priming treatment was significantly better than that of hydro-priming, corroborating the fact that Ca^2+^ may play a key role in this process.

Proline is an important osmotic regulator, which maintains osmotic balance *in vivo* by increasing cell osmotic potential under osmotic stress (Reddy et al., 2015). This study showed that sorghum mesocotyl and root accumulated more proline under salt stress, which was a self-regulating mechanism of plants in response to salt stress. These results are consistent with the results reported by Mulaudzi et al. (2020). Proline not only plays an important role in regulating osmotic balance, but also acts as a molecular chaperone to prevent membrane damage (Delauney and Verma, 1993). In addition to proline, other osmotic adjustment substances including free amino acids, soluble sugars, and soluble protein content changes showed results similar to that of proline. Heinemann et al. (2020) showed that the accumulation of free amino acids may be the result of proteolysis during osmotic adjustment. Therefore, the accumulation of free amino acids may be an adaptive mechanism for plants in response to osmotic stress. Seed priming can effectively enhance this physiological adaptation, which is similar to the results of Islam et al. (2015) who reported that hydro-priming and CaCl_2_ priming increased the proline content, improved salt tolerance, and that the effect of CaCl_2_ treatment was better than hydro-priming. The accumulation of osmotic adjustment substances can maintain a suitable water potential, thus offsetting the adverse effects of osmotic stress caused by increased salt concentration (Kumar et al., 2003). Although the osmotic adjustment substances increased under salt stress, the relative water content in sorghum tissues was low, indicating that the increased osmotic adjustment substances were not enough to alleviate the osmotic stress caused by increased salt concentration. Seed priming further increased the content of osmotic adjustment substances, so that the relative water content in the tissues increased, and the water state was improved.

Ion regulation is another key parameter that reflects the salt tolerance mechanism of plants. Therefore, the evaluation of ion accumulation, especially the accumulation of Na^+^, K^+^, and Ca^2+^ in different plant organs will aid in elucidating the salt tolerance mechanism (Noreen et al., 2010). The decrease in Na^+^ content and the increase in K^+^ content were important reasons for the improvement of plant salt tolerance caused by seed priming (Bajwa et al., 2018). In this study, salt stress increased the K^+^ content in sorghum tissues, because when exposed to salt stress, plants tried to maintain a higher K^+^/Na^+^ in the cytoplasm to maintain ion balance (Shabala and Pottosin, 2014). However, due to the increased accumulation of Na^+^ in tissues under salt stress and the limited ability of plants to absorb K^+^, plants were unable to increase K^+^/Na^+^ through their own ability to regulate ion balance under salt stress. Although hydro-priming has no significant effect on K^+^ content, it reduced the Na^+^ content in the tissues. Therefore, K^+^/Na^+^ was increased, while CaCl_2_ priming influenced both the increase of K^+^ content and the decrease of Na^+^ content. CaCl_2_ priming has a better effect in increasing K^+^/Na^+^ to maintain ion balance under salt stress. Manishankar et al. (2018) reported the important role of calcium signaling in plant K^+^ transport and balance. Therefore, the increased K^+^/Na^+^ concentration under salt stress by CaCl_2_ treatment may be related to the enhanced calcium signaling. Ca^2+^ signaling plays an important role in activating the core SOS pathway by promoting Na^+^ efflux and storage in vacuoles under salt stress (Manishankar et al., 2018). In addition, excess Na^+^ is known to affect the cell wall integrity. The latest evidence showed that the Ca^2+^ signaling plays an vital role in maintaining the integrity of the cell wall and alleviating the inhibition of salt stress on cell growth (Feng et al., 2018). Therefore, Ca^2+^ signal plays a key role in the process of CaCl_2_ priming by improving the growth of sorghum mesocotyl and root under salt stress and probably contributed to better performance of CaCl_2_ priming compared with hydro-priming. In this study, CaCl_2_ priming increased Ca^2+^/Na^+^ by more than three times in mesocotyl, and by nearly two times in roots. CaCl_2_ priming showed a stronger effect in regulating Ca^2+^/Na^+^ than in K^+^/Na^+^ in response to salt stress. Hydro-priming had similar results, but it was inferior to CaCl_2_ priming in regulating ion balance. Plants has several classes of Na^+^ transporters called salt overly sensitive 1 (SOS1), Na^+^/H^+^ exchangers (NHX), and high-affinity K^+^ transporters (HKT). Similarly, there are several K^+^ transporters reported, including class I HKT, K^+^ uptake (KUP), and high-affinity K^+^ transporter (HAK) transporters (Saddhe et al., 2021). Plants use Na^+^ antiporters to transport Na^+^ to apoplasts or vacuoles to maintain low levels of Na^+^ in the cytoplasm. K^+^ transporters are responsible for the absorption and transport of K^+^ (Zhao et al., 2021). In this study, the up-regulation of Na^+^ antiporter genes *NHX2*, *NHX4, SOS1*, and K^+^ transporter genes *AKT1*, *AKT2*, *HKT1*, *HAK1*, and *KUP* expression in the CaCl_2_ priming treatment may be responsible for the decrease of Na^+^ content and the increase of K^+^ content in tissues.

Plants adapt to salt stress not only via osmotic regulation and ion balance adjustment, but also through antioxidant enzyme defense system to remove ROS produced by salt stress (Genisel et al., 2015). There are many reports that indicate that seed priming improved the salt tolerance of plants by enhancing the antioxidant enzyme system (Alves et al., 2020; Jiang et al., 2020). However, studies have shown that seed priming resulted in a partial decrease in the activity of antioxidant enzymes, but the level of reactive oxygen remained low. This is because the priming agent itself had the function of scavenging ROS, leading to a reduction in the anti-oxidation system activation (Genisel et al., 2015). The regulation of ROS metabolic homeostasis is critical for plant responses to abiotic stresses (Sheteiwy et al., 2021; Yang et al., 2021). In this study, seed priming treatment did not increase the activity of the antioxidant enzyme system, however, it decreased their activity. It should be noted that the priming agent used in this study had no effect on reactive oxygen scavenging. Therefore, the reduction in reactive oxygen level is mediated by the priming agent and not the antioxidant enzymes. The down-regulation of antioxidant enzyme genes could be attributed to the decrease in antioxidant enzyme activity. In addition, the balance between production and removal of reactive oxygen was less affected by salt stress due to seed priming, so the reactive oxygen level was lower in CaPS than in the control plants, and the stronger antioxidant enzyme system was not activated. Yang and Guo (2018) suggested that salt stress induced ion stress, osmotic stress, and secondary stress, especially oxidative stress. Oxidative stress was mainly caused by the excessive accumulation of ROS. Therefore, the accumulation of ROS may be related to the degree to which plants are subjected to ion stress and osmotic stress. However, seed priming is a seed pretreatment process carried out before the occurrence of stress. The reduction of reactive oxygen content caused by seed priming may be related to its enhanced osmotic adjustment ability and ion balance. The results of this study also confirmed to this conjecture. Moreover, the change of MDA content also synchronously confirmed that the degree of lipid peroxidation under seed priming treatment was low, but further studies should be conducted to explore the mechanisms of reactive oxygen species production under salt stress and their relationship to osmotic and ion balance regulation in plants.

## Conclusion

**FIGURE 10:**
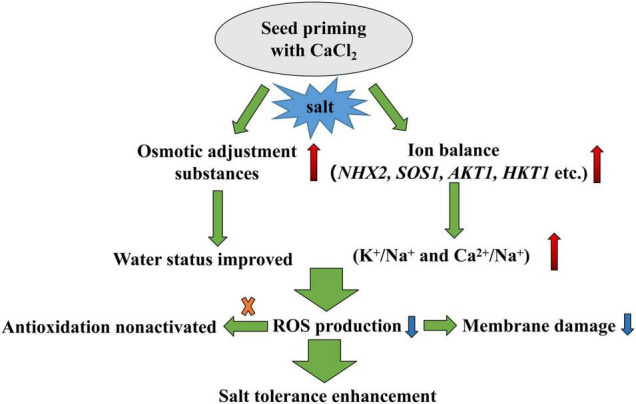
A flowchart of CaCl_2_ priming enhancing salt tolerance during sorghum germination. The red arrows indicate increase and blue arrows indicate decrease.

In conclusion, this study revealed that CaCl_2_ priming increased the content of osmotic adjustment substances in mesocotyl and root of sorghum under salt stress compared with hydro-priming. CaCl_2_ priming regulated ion balance under salt stress by increasing K^+^/Na^+^, especially Ca^2+^/Na^+^. CaCl_2_ priming enhanced the salt tolerance during seed germination and alleviated the inhibition of salt stress on sorghum seed germination and growth (Figure 10). Ca^2+^ played an important role in improving the salt tolerance of sorghum induced by CaCl_2_.

## Data Availability Statement

The datasets presented in this study can be found in online repositories. The names of the repository/repositories and accession number(s) can be found in the article/Supplementary Material.

## Author Contributions

XC performed the experiment, analyzed the data, and drafted the manuscript. YZ conducted the experiment and modified the manuscript. RZ and BL helped in performing the experiment. TC helped in modifying the manuscript. BC provided the resources. All authors contributed to the article and approved the submitted version.

## Conflict of Interest

The authors declare that the research was conducted in the absence of any commercial or financial relationships that could be construed as a potential conflict of interest.

## Publisher’s Note

All claims expressed in this article are solely those of the authors and do not necessarily represent those of their affiliated organizations, or those of the publisher, the editors and the reviewers. Any product that may be evaluated in this article, or claim that may be made by its manufacturer, is not guaranteed or endorsed by the publisher.
